# Incorporating animal-assisted therapy in mental health treatments for adolescents: A systematic review of canine assisted psychotherapy

**DOI:** 10.1371/journal.pone.0210761

**Published:** 2019-01-17

**Authors:** Melanie G. Jones, Simon M. Rice, Susan M. Cotton

**Affiliations:** 1 Orygen The National Centre of Excellence in Youth Mental Health, Parkville, Victoria, Australia; 2 The University of Melbourne, Parkville, Victoria, Australia; 3 Lead The Way Institute, Boronia, Victoria, Australia; Norwegian University of Science and Technology, NORWAY

## Abstract

**Introduction:**

As interest in Animal-Assisted Interventions (AAI) grows, there is increasing need to differentiate informal activities from formal and professionally directed therapies, including mental health focussed Canine-Assisted Psychotherapy (CAP). There have been no reviews focusing exclusively on CAP and the distinct developmental period of adolescence. The aims of this study were to identify the characteristics of CAP interventions, their impacts and their acceptability, tolerability and feasibility for adolescents with mental health disorders.

**Method:**

A systematic review identified studies incorporating canines into mental health treatments for adolescents aged 10–19 years. Studies reporting qualitative or quantitative psychological or psychosocial outcomes were included.

**Results:**

Seven studies were scrutinised. Intervention characteristics varied, including a range of formats, settings, locations, doses, and facilitators. Information on the role of the canines in sessions was sparse. CAP had a positive impact on primary diagnoses and symptomatology, conferring additional benefits to standard treatments for internalising disorders, post-traumatic stress disorder, and equivalent effects for anxiety, anger and externalising disorders. CAP was associated with positive impacts on secondary factors including increased engagement and socialisation behaviours, and reductions in disruptive behaviours within treatment sessions. Global functioning also improved. There was insufficient evidence that CAP improved factors associated with self-esteem, subjective wellbeing, or coping. Good attendance and retention rates indicated high levels of acceptability. Moderate to high tolerability was also indicated. Feasibility may be limited by additional training and logistical requirements.

**Recommendations:**

We recommend the development of theoretically informed, standardised (manualised) intervention protocols that may subsequently form the basis of efficacy and effectiveness testing. Such protocols should clearly describe canine-participant-facilitator interactions via a formalised nomenclature; *spontaneous* (animal-led), *adjunctive* (facilitator-led), and *experiential* (participant-led).

**Conclusions:**

There is emerging evidence to suggest that CAP improves the efficacy of mental health treatments in self-selected adolescent populations via reductions in primary symptomatology, and via secondary factors that improve therapeutic processes and quality, such as engagement and retention.

## Introduction

### Defining animal-assisted interventions

The incorporation of animals into human treatments has a long history. As the field has evolved, much work has been done to both define and describe the nature and types of these interventions, and to explore their efficacy (the ability to produce the desired or intended result within a controlled research context) and effectiveness (how the treatment works in real-life practice) [1, 2]. Some of the earliest attempts to define formal and informal interventions that incorporate animals were made in the 1980s and 1990s by the Delta Society (now Pet Partners) USA. These definitions are possibly the most widely cited, differentiating structured therapies referred to as animal-assisted therapy (AAT) from informal interventions called animal-assisted activities (AAA) [3].

Given the crucial importance of clear definitions to establishing efficacy, considerable work has been done by researchers and international bodies to highlight the need for common terminology [4–6]. A number of key points appear consistent in the existing literature and will be accepted as definitions for the purposes of this paper. First, animal-assisted interventions (AAI) is the umbrella term that refers to the deliberate and meaningful inclusion of animals into human health, wellbeing, or educational interventions. Second, AAAs are less-formal interventions that aim to improve human wellbeing but are not necessarily individualised or documented. Goals may be general and applied to a wide range of target groups equally. Those delivering the intervention need not be licensed professionals but are still trained and/or certified to work safely together with the animal. Third, AAT is a goal-directed and individualised treatment that is measured and documented. AAT is delivered or directed by a qualified or licensed health/human-service professional, within the scope of their professional practice. The human and the animal are trained and/or certified to work safely together to deliver the interventions. The professional may be the animal handler (triangle model) or there may be a professional plus an animal and handler team (diamond model).

This review is particularly focused on mental health specific interventions that focus on psychological and psychosocial outcomes. These are sub-sets within AAT, including, for example AAT in counselling (AAT-C) [7] animal-assisted psychotherapy (AAP) [8] and animal-assisted play therapy (AAPT) [9]. These interventions include human-animal interaction in addition to, or combination with, a recognised and professional form of mental health therapy. The unique relationships formed between humans and animals are seen as key agents of change and are respected in a similar way to the therapist-client relationship. For the purposes of this paper, AAP refers to a sub-set of AAT, focussed on mental health outcomes, and utilising psychotherapeutic techniques or theories. Canine-assisted psychotherapy (CAP) refers to AAP interventions assisted by the intentional and meaningful inclusion of canines.

### Impact of AAI’s on psychological and psychosocial variables

A number of literature reviews have been conducted to explore the efficacy of AAI’s on psychological and psychosocial variables. Eight systematic reviews have indicated that multi-species AAIs may be effective as an adjunctive treatment for human health and wellbeing [10–17]. Across all of the studies reviewed, the most commonly employed animals were canines and equines. Of the seven reviews that explored psychological variables, all concluded that AAI may be beneficial in reducing psychological distress, including depression, anxiety, trauma symptoms, mental illness or addiction [10–16]. Three reviews examined psychosocial variables, all of which concluded that AAI may improve behaviour, communication or social skills, especially in Autism Spectrum Disorders (ASD) [12, 15, 17]. In all cases, these findings are to be interpreted conservatively given the variability and poor methodological quality of the literature.

Although a there are a number of reviews that have focused specifically on equine-assisted interventions [18–23], no such reviews exist for canines. This adds to the difficulties assessing the impact of canine-assisted interventions. Similarly, whilst there have been reviews of the impact of AAI’s on children and young people [15, 20, 22, 24, 25], most reviews have examined AAIs across the lifespan [10, 11, 13, 14, 18, 19, 21, 26, 27]. We could find no reviews focused on the distinct developmental period of adolescence (10–19 years) [28].

### AAI versus AAT/P

Whilst the aforementioned reviews all examined psychological and or psychosocial variables, only one focused specifically on AAT, exploring the impact of AAP on trauma-related symptoms across the lifespan [14]. In the majority of reviews, the authors failed to delineate simple animal-presence, from therapeutic mental health treatments that incorporated animals. Without these clear distinctions, it is virtually impossible to establish an evidence-base for the different types of AAI’s [6, 29]. In all but Germain, Wilkie [14] review, the authors compared very brief unstructured interactions, often facilitated by a volunteer handler, with comprehensive therapies, such as group counselling facilitated by a trained therapist and animal, in order to draw conclusions about how effective AAT is in treating certain mental health (or other) conditions. Kamioka, Okada [10] defined AAT so broadly as to compare brief unstructured interactions, structured dairy-farm work, goal-focused therapeutic interventions, living with a companion bird and living with a service canine. Hoagwood, Acri [15] stated their definition was AAT, but the literature-review also included unstructured, volunteer-based interactions rather than therapy “*directed by health and human service providers as part of their profession”* [30] and “*within the scope of the professionals’ practice”* [5]. Specifically, there were no psychotherapeutic techniques or theories incorporated into the therapy. None of these reviews were species-specific, including a broad range of animals such as equines, canines, felines (cats), farm animals and birds. Consequently, there is no literature synthesis and hence no current consensus on the efficacy or effectiveness of canine inclusion in mental health treatments or psychotherapy.

### The inclusion of canines in psychotherapy (CAP)

Individual studies exploring the impact of adding a canine to psychotherapy, show that psychological and psychosocial variables may be improved over and above psychotherapy without a canine (treatment as usual) for a range of populations. Children and young people with ASD in residential care had significantly fewer interpersonal and functional problems [31] and children with ADHD participating in counselling displayed reduced symptomatology [32]. Children participating in a trauma treatment group showed significant reductions in PTSD symptoms of intrusion, arousal, avoidance and dissociation [33]. Adults participating in a trauma treatment analogue experienced significant reductions in distress and depressive symptoms [34]. Adult inpatients diagnosed with schizophrenia had reduced anhedonia [35] and other negative symptoms [36]. Adults hospitalised for substance dependence demonstrated increased therapeutic alliance [37] and interpersonal socialisation [38]. Women with breast cancer attending counselling reported positive anticipation towards sessions, and therapeutic benefits such as reduced distress, and the promotion of feelings of peace and calm [39].

A number of these studies reported that the presence of canines had a direct impact on the primary diagnoses and clinical symptomatology of the participants [31–36]. These were factors that were specifically targeted by the intervention. Other studies reported that the presence of canines improved secondary factors, including therapeutic processes, or outcomes that were of benefit to participant wellbeing, but were not necessarily the primary focus of the intervention. Significant improvements have been seen with therapeutic alliance [37] engagement [39], interpersonal socialisation or trust [38], acute anxious arousal (distress) [34, 39], and functioning [31].

In each of the studies cited above there was some variation in how canines were integrated into the therapeutic process from structured activities [31, 32, 35, 36], to natural and spontaneous responses to the mood or behaviour of the participants [34, 37, 39]. Few studies explored the nature of the canine’s engagement, temperament or behaviour on clinical outcomes. In contrast, two studies [40, 41] indicated that this may be a crucial component of the treatment process. Firstly, a structured psychotherapy and psychoeducation group that included a calm and gentle Collie was not as effective at reducing depression in adult college students as a ‘non-directive’ group *without* psychotherapy, that focussed on the behaviour of an engaging, energetic and ‘puppy-like’ Collie [40]. Secondly, in trauma treatment groups for children and young people aged 7–17 years who had experienced sexual abuse [41], the inclusion of 15 minutes of unstructured canine contact at the beginning of sessions (preceded by 30 minutes in the waiting room) resulted in significant reductions in trauma symptoms. In phase two, the therapists noted that it was difficult to transition from the canine visit to the group therapy content. Consequently, therapeutic stories were developed to both integrate the presence of the canine, and aid with the transition to group therapy once the canine left the session. This resulted in further significant improvements to trauma symptoms over and above the groups with canines but no stories, and over treatment as usual (without canines) [41]. This finding is consistent with previous reports that the meaningful inclusion of a canine via storytelling is an effective tool for children who have experienced sexual abuse [42, 43]. Taken together, these studies highlight the need to explore the role of canines within the treatment process, and to establish how meaningful the role of the canine is to the participants.

### AAI for adolescents

There is some evidence to suggest that AAT, and in particular CAP, is an effective intervention for child and adolescent populations with neurodevelopmental difficulties [31, 32] and trauma symptoms [33, 41]. Adolescence, however, is a distinct developmental period for a number of reasons, including reorganisation of regulatory systems in the brain [44], increasing importance of the peer-group and appropriate social experiences [45], increasing emotional and cognitive demands, sexual and identity development and individuation [46]. Importantly, many mental health disorders have their origin in childhood and adolescence [47], and mental health difficulties during adolescence can severely impact educational and social functioning, causing severe distress [48]. Indeed, the negative sequelae of mental health problems have been identified as the leading contributor to the burden of disease among young Australians [49]. Despite this, young people have historically been a difficult population group to engage. Reports on the nature of youth mental illness consistently state that adolescents are concerned about the stigma associated with mental illness, and express concerns about ‘talking with strangers’ which may negatively impact seeking professional help [48, 50].

There is a need to make interventions for this age group engaging in order to overcome the possible stigma associated with help seeking; mental health interventions and services need to be ‘youth friendly’ and ‘appealing’ [50]. AAIs have been repeatedly shown to be an effective tool to assist with engagement and rapport building [9, 37, 51, 52]. Animal presence makes strangers and therapists seem more trustworthy and makes people feel more safe and comfortable disclosing [53–55]. These findings provide some support for anecdotal claims that AAIs are both acceptable and well tolerated by adolescents (e.g. [56]). Despite this, acceptability and tolerability have rarely been directly assessed in the literature [57, 58].

It is therefore important to understand how acceptable adolescents find mental health interventions that incorporate canines. Acceptability refers to a person’s experience of the intervention, and can be assessed by gathering drop-out or cessation data [59] in combination with qualitative sampling [60]. Willingness to enrol and participate can also be interpreted as measures of perceived acceptability [58]. Tolerability is frequently used in the context of health risk-management (e.g., how well is a medication tolerated) and refers broadly to adverse or unintended consequences arising from the intervention, versus intended outcomes [61]. Whilst adverse or unintended consequences have been described in some AAI studies, there is a general lack of information about tolerability [57]. Working with animals also raises questions of feasibility. In a clinical context, feasibility refers to the extent to which an activity is “*physically*, *culturally or financially practical or possible within a given context”* [62]. There are also additional logistical, financial and training requirements for both the humans and animals in AAI, prompting calls for greater reporting of constraints such as external limitations in AAI research [63].

### Objectives and research questions

The overall purpose of this systematic review was to explore the impact of CAP for young people in the distinct developmental period of adolescence, as defined by the WHO [28], aged 10 to 19 years. The aims of the study were threefold, to identify: (i) the characteristics of the interventions (description of the intervention including; activities, underlying psychological theories, the role of the canine(s) in treatment, and the role of the facilitator(s) in treatment; and replicability); (ii) the impacts of CAP for adolescents with mental health disorders on primary (clinical diagnosis and symptomatology) and secondary factors (factors of benefit to participant wellbeing or the therapeutic process); and (iii) the evidence with regard to the acceptability, tolerability and feasibility of CAP for adolescents with mental health disorders.

## Methods

### Study design

A comprehensive database search was conducted to identify goal-focused CAP aimed at improving mental health outcomes for adolescents 10–19 years, and in accordance with the Preferred Reporting Items for Systematic Reviews and Meta-Analyses (PRISMA) [64] (S1 Fig).

#### Eligibility criteria

Studies were eligible for inclusion in the analysis if they met the following criteria:

- Treatment-based (goal-focussed) interventions, utilising psychotherapeutic techniques or theories, facilitated by, or under the direct guidance of, mental health professionals;
- Including canines (irrespective of the nature of the interactions), but no other species; and
- Literature published in peer reviewed publications, written or translated into English and reporting qualitative or quantitative results to mental-health outcomes.

### Search strategy

The search protocol was designed to be inclusive and broad in order to capture the vast array of terminology utilised in the field. Previous systematic reviews of AAT were consulted as a guide [10, 15, 26]. A search strategy was developed in consultation with a specialist research librarian. Search terms were identified as outlined below. Where discrepancies occurred, all authors were consulted until consensus was reached. Given the poor methodological quality of existing studies in the field, only those studies which had undergone peer review and publication were accepted.

#### Databases

The following databases were searched: PsycINFO; PubMED; Scopus; and MEDLINE. No date range was stipulated. The following search terms were utilised in the PsycINFO search, and all other databases followed an equivalent search protocol utilising the same terms (S1 Table).

1. ((animal or dog* or canine or non-human or pet or human-animal or animal-assisted) not rat* not rodent* not equine* not horse* not mice not mouse not ape* not monkey* not fish not bird not chimpanzee* not animal model* not animal stud* not chicken* not animal find*).mp. [mp = title, abstract, heading word, table of contents, key concepts, original title, tests & measures]
2. (counselling or counseling or intervention or learning or psychotherapy or therap* or mental* or emotion* or behaviour* or behavior* or affect* or empath* or trauma*).mp. [mp = title, abstract, heading word, table of contents, key concepts, original title, tests & measures]
3. (youth or young person or teen* or adolesc* or child*).mp. [mp = title, abstract, heading word, table of contents, key concepts, original title, tests & measures]
4. 1 and 2 and 3

#### Reference searching

The reference sections of included papers were hand searched by the lead researcher (MJ) for additional studies missed by the database searches. Relevant titles were identified and cross-checked for duplication. Remaining titles were searched, and abstracts reviewed in line with the search strategy. Studies identified for full-text review were downloaded.

### Data extraction

For each identified study, a range of data were extracted including: study characteristics and design; participant, facilitator and animal characteristics; intervention characteristics; and outcomes and conclusions.

Studies were assessed for methodological quality using the National Institute of Health (NIH) guidelines for quantitative studies [65, 66] and the Critical Appraisal Skills Program (CASP) for qualitative research [67, 68]. Each study was rated ‘good’, ‘fair’ or ‘poor’ for methodological quality. Studies were further ranked in accordance with Australia’s National Health and Medical Research Council (NHMRC) Evidence Hierarchy for Interventions [69, 70]. This tool ranks intervention studies according to the quality of their research design to manage bias and confounders, and the degree of confidence that can be attributed to the resulting conclusions (see Table 1).

**Table 1:** Designations of levels of evidence [70].

Level of Evidence	Study design
I	Evidence obtained from a systematic review of all relevant randomised controlled trials
II	Evidence obtained from at least one properly-designed randomised controlled trial
III-1	Evidence obtained from well-designed pseudorandomised controlled trials (alternate allocation or some other method)
III-2	Evidence obtained from comparative studies (including systematic reviews of such studies) with concurrent controls and allocation not randomised, cohort studies, case-control studies, or interrupted time series with a control group
III-3	Evidence obtained from comparative studies with historical control, two or more single arm studies, or interrupted time series without a parallel control group
IV	Evidence obtained from case series, either post-test or pre-test/post-test

A description of the participants was extracted, including age and sex. Diagnoses or difficulties were reported if identified by the authors (e.g., depression, anger). Facilitators were accepted as mental health professionals if they could be identified either by qualification, job title or job role. Descriptions of canines were extracted, including information about their training, certification or evaluation.

Details about the intervention, such as program outlines or session activities, were reported as a measure of standardisation or replicability. The nature of engagement between participants and canines was retrieved and coded as structured (facilitator-directed) interactions, semi-structured interactions, and/or spontaneous interactions. The format of interventions was coded as group or individual. The setting of the intervention refers to the location in which the intervention occurred, including information about indoors versus outdoors where available. The dose for each intervention included frequency (e.g., weekly) and total hours, calculated by adding the duration of each session (e.g., 60 minutes), and the length of the intervention (e.g., 12 sessions).

Outcomes and conclusions were extracted, including information about the type of assessments used and a summary of the results. Information about the acceptability, tolerability and feasibility were extracted. Limitations, future directions and conclusions drawn by the authors were all documented.

## Results

**Fig 1:**
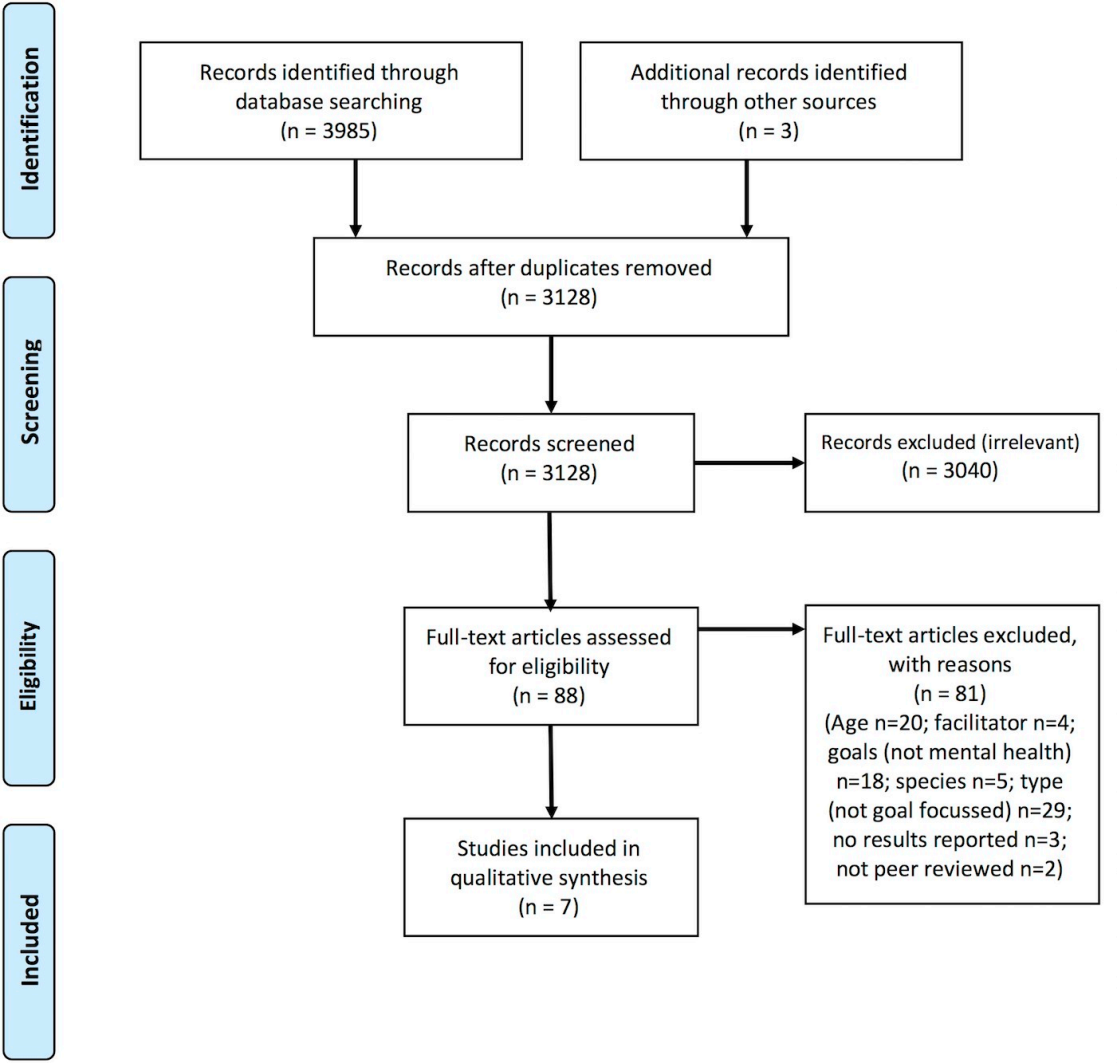
PRISMA flow diagram documenting studies included in the review.

A total of 3985 studies were identified for initial screening via database searching. After exclusions (see Fig 1), 88 studies were selected for full-text screening, including 3 studies identified via hand searching of reference lists. Seven studies met the inclusion criteria and were included in the final analysis. These seven studies examine a range of presenting issues, including mood, anxiety, trauma, anger and disruptive behaviour, self-concept, adaptive functioning and global functioning, and clinical severity of serious psychiatric illness. A range of assessment tools were used by the authors, including therapist report, youth self-report, observation and qualitative thematic analyses. Given the heterogeneity of the methodologies, assessment tools and presenting issues, meta-analysis was not feasible.

### Studies identified

All seven of the studies were published in the last 17 years, with four published in the last 5 years. Three of the studies were conducted in the USA, two in Italy, one in South Africa, and one Israel. All studies had the stated goal of reducing the impact of psychological distress or disorder, and/or to improve the adaptive functioning of the client (see Table 2).

**Table 2:** Study characteristics and design.

No.	1^st^ Author	Year	Intervention Goals	Design	Sample Size	Quality	Level of evidence
**1**	**Hamama, L.** (Israel)	2011	To reduce the psychological distress (i.e. depression & PTSD symptoms) and improve psychosocial functioning (self-confidence & subjective wellbeing) among teenage girls who were exposed to physical or sexual abuse in group counselling incorporating a canine	Pre-post longitudinal design, and a cross-sectional design comparing I to C	I = 9 C = 9	Fair	III-2
**2**	**Hanselman, J. L.** (USA)	2001	To reduce violent behaviours including the prevention of animal abuse, and increase pro-social behaviours, empathy & self-awareness during an Anger Management Group incorporating a canine	A-B-A design where A = baseline (before); B = intervention (during) and A = outcome (after)	I = 7	Poor	IV
**3**	**Hartwig, E.** (USA)	2017	To increase self-concept, and decrease anxiety, depression, anger and disruptive behaviour in youth with emotional and psychosocial difficulties in individual therapy with a canine, over and above therapy without the canine.	Randomized comparison group design	I = 15 C = 14	Fair	III-1
**4**	**Lange, A. M.** (USA)	2007	To explore the experiences of adolescents in an anger management counselling group that included a canine	Exploratory study using qualitative interviewing and observations	I = 5	Poor	IV
**5**	**Lubbe, C.** (South Africa)	2013	To retrospectively analyse the role of a canine in individual counselling for a traumatized adolescent with a history of family dysfunction	Qualitative interview and (retrospective) document analysis	I = 1	Fair	IV
**6**	**Stefanini, M. C.** (Italy)	2015	To improve clinical outcomes (global functioning, self-perceived internalizing and externalizing problems) and observed behaviour patterns for psychiatric inpatients involved in individual and group sessions with a canine, over and above standard hospital treatment	Pre-post experimental design with RCT	I = 17 C = 17	Good	II
**7**	**Stefanini, M. C.** (Italy)	2016	To improve self-perceived emotional and behavioural symptoms, clinical outcomes, and observed behaviour patterns in adolescents with acute psychiatric disorders involved in individual and group sessions with a canine, over and above standard hospital (inpatient) treatment (TAU).	Pre-post experimental design with RCT	I = 20 C = 20	Good	II

I = Intervention group or experimental group, C = Control or Comparison group, RCT = Randomised Controlled Trial; PTSD = Post Traumatic Stress Disorder; TAU = Treatment as Usual

### Study design and methodological quality

A range of methodological designs were reported, including three randomised controlled trials (RCT), one case-controlled study, one pre-post design and two qualitative studies. Only two studies had ‘good’ methodological quality, both were RCTs [71, 72]. In both studies, computer-generated randomisation was employed, and equivalence of experimental and control groups was reported. Stefanini, Martino [71] reported blinding of assessors and use of independent observational measures. Stefanini, Martino [72] utilised a mix of self-report, staff-report and independent observation measures. In both cases, the authors reported a primary focus on methodological rigour.

Three of the studies [56, 73, 74] were ranked as ‘fair’ for methodological quality, indicating the possibility of bias. Hartwig [73] reported randomisation, however, the process was not described. Pre-intervention data was not reported, making comparison between groups difficult, and only self-report outcome measures were used. Hamama, Hamama-Raz [74] reported the possibility of bias in the participant selection process, acknowledging they were selectively identified by the school counsellor. Subjective self-report tools were used alongside the validated tools, reducing the overall confidence in the conclusions obtained. Lubbe and Scholtz [56] reported on a single case-study, using qualitative methodologies integrating information from multiple sources over the duration of treatment, plus at follow up 18 months later. The authors directly attributed a number of results to the presence of the canine, despite an acknowledgment that confounding factors were not explored (e.g., the role of the therapy, relationship with the facilitator, outside relationships and age/development).

Two of the studies were ranked as ‘poor’ for methodological quality [75, 76]. Lange, Cox [75] acknowledged that their sample size was too small to draw any conclusions from the data. The process for recruiting and selecting participants was not reported, nor the reason for 2 of the 5 participants withdrawing from the study (i.e., being ‘unavailable’ for the follow up interview). It was also unclear if the facilitator conducted the qualitative interview, which has the potential for bias. Hanselman [76] report of treatment goals within the paper was very detailed; but there were insufficient details on participant eligibility, screening, and statistical analyses to assess the outcomes reported. There was also heavy reliance on subjective (non-validated) self-report measures, and informal facilitator observations, in many of the author’s conclusions.

### Participant, facilitator & canine characteristics

Four studies reported interventions for participants aged 13–16 years, whilst three studies involved participants from the wider developmental period 10–18 years or 11–17 years (see Table 3). Narrower age ranges appear have been utilised for group intervention programs in which participants worked collaboratively together and would ideally be developmentally matched [74–76]. The majority of interventions were conducted with male and female participants except Lubbe and Scholtz [56] (a single case study with 14 year old male) and Hamama, Hamama-Raz [74] (a group program for females).

**Table 3:** Participant, facilitator and animal characteristics.

No.	1^st^ Author	Description of Participants	Age	Sex	Facilitators	Canines
**1**	**Hamama, L.**	• Intervention group: teenagers from a selected high school, identified by the school counsellor and having experienced physical or sexual abuse 3–4 years prior, low achievements in school and interpersonal difficulties, who consented to attend. • Comparison group: matched by school, age, without a history of abuse or interpersonal difficulties	14–16 years	F = 18	• Facilitators—Two social work students. • Supervisor–Master Social Worker and animal-assisted therapist	Canines that were matched to each participant
**2**	**Hanselman, J. L.**	Adolescents self-selected or court mandated to attend an Anger Management Group who were interviewed (screened) for intellectual ability and age.	14–16 years	F = 2 M = 5	Facilitators– 2 counsellors	Two canines
**3**	**Hartwig, E.**	Children referred for counselling by parents, schools or agencies for emotional issues. Presenting issues were varied, including grief, loss, anxiety, depression and self-concept in the contexts of school, home and family, and who consented. Participants were screened and excluded for fears, allergies or history of abuse to animals	10–18 years	F = 16 M = 13	• Facilitators—Professional and student counsellors who had completed Pet Partners Handler Course • Supervisor–Professional counsellor with interest and training in animal assisted therapy	Counsellors own canines, assessed as appropriate through training and assessment, and evaluated by Pet Partners as suitable to work in complex environments
**4**	**Lange, A. M.**	Adolescents who consented to attend an anger management group	13–16 years	F = 2 M = 3	Facilitator–an experienced counsellor with an interest and training in animal assisted therapy	A specially selected canine assessed for temperament and obedience by Therapy Canines International evaluators and vet checked.
**5**	**Lubbe, C.**	A boy who attended therapy after being admitted to a place of safety by his mother. He had a history of school refusal, and facial scarring from a benzene bomb injury when young.	14 years	M = 1	Facilitator—Masters level student in Educational Psychology	One small canine
**6**	**Stefanini, 2015**	Children and adolescents with a psychiatric diagnosis who were patients (for 2 weeks to 3–4 months) at the acute psychiatry unit of the Meyer Pediatric Hospital. ICD-9 diagnoses included mood disorders, schizophrenia, anxiety disorders, and eating disorders	11–17 years	F = 9 M = 8 Per condition	• I = Facilitators–staff member, animal-handler trained in AAT • I & C = TAU hospital staff	Canines examined by vet to Pet Partners sanitary protocol
**7**	**Stefanini, 2016**	Children and adolescents with severe psychiatric diagnoses who were inpatients (for 2 weeks to 3–4 months) of the Child and Adolescent Psychiatric Unit of the Meyer Pediatric Hospital. ICD -10 diagnoses included mood disorders, anxiety disorders and eating disorders.	11–17 years	F = 11 M = 9 Per condition	• I = Facilitators–registered psychologist, animal-handler trained in AAT • I & C = TAU hospital staff	Canine and Handler teams matched to each participant, certified with National School for Guide Canines for the Blind, and vet checked

F = female, M = male; I = intervention group or experimental group, C = control or comparison group; ICD-9/10 International Classification of Diseases Ninth revision/tenth revision; TAU = Treatment as Usual

Participants presented with a range of difficulties, including: females with a history of physical or sexual abuse, low achievement in school & interpersonal difficulties [74]; males and females requiring an anger management intervention [75, 76]; adolescents referred to counselling for emotional issues [56, 73]; or adolescents admitted to hospital with severe psychiatric illness [71, 72]. Diagnostic information at intake was provided only by Stefanini, Martino [71] and Stefanini, Martino [72], and the cohort for each study was independent.

Facilitators were reported to be qualified or student counsellors [73, 75, 76], social workers [74], psychologists [72], or psychiatric hospital staff [71]. All but two of the studies reported that facilitators were explicitly trained or supervised in AAT [56, 76]. Two studies also reported employing animal-handlers [71, 72].

Four of the studies included information about the selection, assessment or certification status of the canines employed [71–73, 75]. None reported details about the canine’s breed/type or size, although Hartwig [73] did indicate that the canine’s temperament or behaviour may impact outcomes. Lubbe and Scholtz [56] included photos in the paper, showing a small canine similar in appearance to a Yorkshire Terrier. Hanselman [76] reported employing two canines but provided no other information. The number of canines employed in the interventions was not reported by Hamama, Hamama-Raz [74] or Hartwig [73]. Stefanini, Martino [71] and Stefanini, Martino [72] reported that five canines and their handlers were employed.

### Intervention characteristics

A broad range of interventions were employed in the reviewed studies, including individual counselling, group counselling, and combined interventions in a variety of settings such as a school, hospitals and community settings (see Table 4).

**Table 4:** Intervention characteristics.

No.	1^st^ Author	Intervention Description	Nature of human-animal interaction	Theoretical underpinnings	Format	Setting	Dose
**1**	**Hamama, L.**	• I = Goal-focused canine-related activities such as building trust, training, talking to or walking the canines • C = psychosocial topics such as trust and confidence building exercises • Brief program outline & session examples provided	Structured and semi-structured interactions	To increase trust and socialisation via the presence of animals, and to facilitate role changes via canine-related activities.	Group	Secondary School setting • I = classroom & local park • C = classroom	3 hours once per week for 12 weeks. Total hours = 36
**2**	**Hanselman, J. L.**	• I = Anger management group incorporating creative and projective activities, a Scared Straight night, and discussions while canines were present • Detailed intervention goals provided. • No program/session outlines provided	Spontaneous	Cognitive Behavioural Therapy; Attachment Theory; To lower client arousal and anger via the presence of animals	Group	Not described	10 sessions over 12 weeks
**3**	**Hartwig, E.**	• HART model intervention with (I) or without(C) canines. Integrates creative and expressive techniques into therapy. Standardised intervention to be applied by multiple therapists. • Example activities provided.	Structured interactions	Solution Focused Therapy; Play therapy; To improve engagement and motivation via the presence of an animal	Individual	Community counselling service (small indoor counselling rooms)	50-minute sessions weekly for 10 sessions. Total hours = 8.3 (Plus caregiver consults)
**4**	**Lange, A. M.**	• I = Anger management group incorporating pre-determined goal-focussed activities with the canine, and information about the care and keeping of an animal • No program/session outlines provided	Structured and spontaneous interactions	That the presence of an animal may provide benefits to the participants based on previous research	Group	Not described	Not described
**5**	**Lubbe, C.**	• I = individual counselling with a canine, therapeutic goals identified • No program/session outlines provided.	Structured, semi-structured and spontaneous interactions	That the presence of the animal may assist with engagement and rapport as previous therapy had not been successful	Individual	Counselling service indoors	Not described
**6**	**Stefanini, 2015**	• I = Hospital TAU plus structured goal-focussed sessions incorporating a canine, engaging in a range of activities such as play, physical contact, grooming, cleaning, basic obedience, walking and agility • C = Hospital TAU • Brief example session activities provided.	Structured and semi-structured	To build the evidence base that AAT may assist with the treatment of psychiatric illness.	Individual & group	Psychiatric Hospital activity room (indoors) and garden (outdoors)	45-minute sessions weekly for 3 months. Total hours = 9
**7**	**Stefanini, 2016**	• I = Hospital TAU plus structured goal-focussed sessions incorporating a canine, engaging in a range of activities such as play, physical contact, grooming, cleaning, basic obedience, walking and agility • C = Hospital TAU • Brief example session activities provided.	Structured and semi-structured	To incorporate an animal in TAU to enhance trust, improve therapeutic alliance & therapeutic process	Familiarisation & matching; 5 individual and 5 group sessions	Psychiatric Hospital activity room (indoors) and garden (outdoors)	45-minute sessions weekly for 3 months. Total hours = 9

I = intervention group or experimental group, C = control or comparison group; TAU = Treatment as Usual

One intervention was reportedly standardised; the HART model developed by Hartwig [73]. This was a 10-week (8.3 total hours) intervention based on the concepts of solution focused and play therapies, and utilising creative and expressive techniques delivered during individual counselling sessions. The control group engaged in the standard HART curriculum, whilst the intervention group engaged in a HART plus AAT curriculum, where session activities were modified to incorporate a canine. Examples of session activities were provided, and the participant-canine interactions appeared to be structured (facilitator-directed). There were multiple facilitators (trained counsellors) and canines employed during the intervention, with AAT facilitators working with their own certified therapy canines. Lubbe and Scholtz [56] reported a single case study; an individual counselling intervention, facilitated by a counsellor and her own canine. The intervention was not standardised, but was delivered in a responsive way to the participant’s presenting issues and treatment goals. Interactions with the canine contained a mix of structured (facilitator-led), semi-structured and spontaneous interactions. The dosage of the intervention was not described. Follow-up interviewing was conducted with the participant 18 months post-treatment. Both individual counselling interventions [56, 73] were delivered indoors, in typical counselling rooms.

The interventions delivered by Stefanini and colleagues [71, 72] consisted of individual sessions (including matching participants with the canines) followed by group sessions over a period of 3 months (9 total hours) at a psychiatric hospital. There was no reported overlap in participants between the two studies. Interventions were conducted weekly, in addition to hospital treatment as usual (TAU). Activities were tailored to the participants’ own treatment goals, including structured (facilitator-led) and semi-structured canine interactions conducted outside in the hospital garden, except when the weather was inclement. The comparison groups received hospital TAU without the AAT intervention.

The remaining three studies reported group interventions [74–76]. Both Hamama, Hamama-Raz [74] and Lange, Cox [75] reported goal-focused activities set out by the facilitators. Hamama, Hamama-Raz [74] provided a programme overview with session examples, indicating a structured and pre-determined intervention. This was the most intensive intervention reviewed, with a total of 36 hours provided over 12 weeks. Canines were matched to each participant. Activities were largely structured and semi-structured, conducted in a local park close to the school. The comparison group engaged in equivalent or similar activities, not including canines, based within the school classroom. The facilitators, two social work students, ran both groups. In comparison, the intervention delivered by Lange, Cox [75] was developed in response to the participants’ goals. The facilitator, a counsellor, developed goal-focused activities after feedback from the participants about their ideas. No session details were made available, but qualitative analysis indicated both structured and spontaneous interactions.

Unlike the other studies, Hanselman [76] reported that two canines were present only for certain periods during the intervention, in particular during group discussions. The intervention was based on the principles of Cognitive Behavioural Therapy (CBT) with adaptations based on attachment theory, and comprised 10 sessions facilitated by two counsellors. It was unclear how many sessions or for what duration the canines were present. Informal observations made by the facilitators indicated largely spontaneous interactions.

### Psychological & psychosocial outcomes

A summary of outcomes reported by each of the authors is outlined in Table 5. The results are categorised by area of intervention focus below.

**Table 5:** Outcomes and conclusions.

No.	1^st^ Author	Assessment type (inc. measures)	Results summary	Feasibility, tolerability acceptability & dropout	Limitations & future directions	Author conclusions
**1**	**Hamama, L.**	Self-report questionnaires PCL-C, CESD; Likert scales (subjective wellbeing and coping with stressful life events)	• I pre to post = Rapid decline in PTSD symptoms, & risk for PTSD diagnosis. No sig improvement to depression or subjective wellbeing or coping. • No sig diffs between I and C by end of intervention on PTSD symptoms	No dropouts evident or reported. Potential subject pool 20 (I)	Small sample size, poss selection bias	• Group therapy contributes to trauma healing. • Sense of control, mastery aided by canines
**2**	**Hanselman, J. L.**	Self-report questionnaires STAS-TAS, CABS, BDI-II; subjective mood thermometers (tension, confusion, fatigue, depression); facilitator observations	• Sig reduction in emotional & behavioural anger; • Sig increase in animal bonding; • Sig increase in depression; • observed improvements in pos. behaviour when canines present	• No dropouts evident or reported. Potential subject pool unknown. • Increase in depression attributed to increased emotional awareness & reduced substance use.	More sessions (12) and longer duration (2h) required for behavioural change. Qualitative data should be increased. Parent feedback should be analysed & more data sought.	The results confirm previous studies that animals are beneficial in treatment.
**3**	**Hartwig, E.**	Self-report questionnaires BYI-II (5 scales)	• No sig diff between I and C on BYI-II. • Sig reduction in BAI, BDI, BDBI but not BANI, or BSCI in both I and C conditions	• No dropouts evident or reported. Potential subject pool unknown. • Requires extensive staff training & supervision, Difficulties incorporating canines into curriculum	• Canine activities not sufficiently experiential. Small sample size. Small counselling rooms. • Explore impact of canine temperament & energy.	HART curriculum produces sig decreases in anxiety, depression, anger and disruptive behaviour. CAT useful adjunct
**4**	**Lange, A. M.**	Structured interview and subsequent qualitative analysis	Participants report canine presence is beneficial for humour, calming, attendance, disclosure, self-soothing, feeling attached	• 2 of 5 participants were not available for the follow up interview. • Therapist training needed. • Canine assessment & liability issues must be addressed.	• Sample size too small for thematic analysis. • Need to explore efficacy with other presenting issues	Potential benefits include calming, humour relief, safety in disclosing, experiences of empathy, motivation to attend.
**5**	**Lubbe, C.**	Document analysis & semi-structured interview for thematic analysis	Five themes identified; facilitating relationship, communication, physical affection, socialisation, and self-esteem.	• Author’s observations and thematic analysis indicate that the client was well engaged, unlike previous counselling attempts. • Requires properly trained animal & handler, so possible limitations to transferability/scope.	Findings may not be attributable to canine presence	Canine presence promotes engagement and facilitates therapeutic process
**6**	**Stefanini, 2015**	Staff reported measures; C-GAS, format of hospital care, ordinary school attendance; and observational/ behavioural coding.	• I = Sig improvement in global functioning, format of care (inc. duration of hospital stay), school attendance over TAU (C). • I = Sig increases in observed in-session behavioural participation, animal interaction & affection, adult & peer socialisations, reduced withdrawal	I = Dropout was zero, attendance 100%. Potential subject pool unknown.	• Small sample size in single location. • Follow up at 6 and 12 months recommended. Analyse results by diagnosis. Explore mechanisms of interaction between patient, animal and operators.	• I = significant clinical and behavioural improvements over TAU. • Animals may act as a catalyst in the therapeutic process, esp. socialization but mechanisms of action require further study
**7**	**Stefanini, 2016**	Staff report C-GAS, and observational/ behavioural coding; YSR	• I = Sig decrease in internalizing probs, sig increase in total competence & global functioning over TAU (C). • I = Sig decrease in externalizing probs pre to post. Not sig compared to TAU (C) (rate of change). • I = Sig increase in observed in-session behavioural participation; interaction & affection with animal; socialization with adults and peers; sig. decrease in withdrawal.	I = Dropout was zero, attendance 100%. Potential subject pool unknown.	• Small sample size in single location. Findings should be replicated in various sites, ages and diagnoses. • Explore mechanisms of action in AAT	Hypothesis supported that AAT reduces emotional & behavioural symptoms and increases global competence and psychological functioning over TAU. AAT may be more effective for internalizing symptoms.

I = intervention group or experimental group, C = control or comparison group; TAU = Treatment as Usual; PTSD = Post Traumatic Stress Disorder

Assessments and Measures

BDI-II = Beck Depression Inventory (second edition)

BYI-II = Beck Youth Inventories (second edition) Including—Anxiety (BAI), Depression (BDI), Disruptive Behavior (BDBI), Anger (BANI), and Self Concept (BSCI).

C-GAS = Children’s Global Assessment Scale

CABS = Companion Animal Bonding Scale

CESD = The short Centre for Epidemiologic Studies Depression Scale

PCL-C = Post Traumatic Stress Disorder checklist–Civilian

STAS-TAS = State-Trait Anger Scale

YSR = Youth Self Report

#### Psychological variables: Post-traumatic stress disorder (PTSD)

Hamama, Hamama-Raz [74] reported a significant decline in PTSD symptoms (*p* = .018) and a significant reduction in the risk for a PTSD diagnosis (*p* = .046) on the self-report measure PTSD Checklist–Civilian (PCL-C) from pre to post-intervention. At Pre-test the intervention group scored significantly higher on the PCL-C than the comparison group who had no exposure to traumatic events (*p* = .014); this difference was not present by the end of the intervention (*p* = .409).

#### Psychological variables: Depression, anxiety and internalising problems

Hamama, Hamama-Raz [74] assessed depression via the short Centre for Epidemiologic Studies Depression Scale (CESD). The intervention group (with trauma history) showed no significant reduction in depression pre- to post-intervention (*p* = .063). The intervention group had significantly higher rates of depression than the comparison group (without trauma history) pre-intervention (*p* = .022) which was not evident post-intervention (*p* = 0.316). Hartwig [73] assessed depression and anxiety using the Beck Depression Inventory (BDI) and Beck Anxiety Inventory (BAI) respectively. Paired comparisons indicated a significant reduction in both depression and anxiety from pre- to post-test for both the intervention group (HART curriculum + AAT), and the comparison group (HART curriculum without AAT) (*p*’s < .05). There was no difference between the intervention group (i.e., AAT) and the comparison group on either measure.

Hanselman [76] assessed depression with the Beck Depression Inventory (BDI-II). Mean scores for depression increased significantly from pre-intervention to post-intervention, with results remaining in the ‘moderate’ range. Various subjective mood states were assessed via mood thermometers for tension, confusion, fatigue and depression at the beginning and end of sessions. In contradiction to the BDI-II scores, the author reported that the mood thermometers indicated an improvement in subjective mood state over the course of the intervention. Statistical analyses were not reported making interpretation of these results difficult.

Internalising symptoms were measured by Stefanini, Martino [72] on the Youth Self Report (YSR). There was a significant decrease in symptoms for the intervention group (TAU + AAT) but not the control group from pre- to post-test (*p* < .05). The intervention group also scored significantly better when compared to the control group (TAU) on rate of change for internalising symptoms (*p* = .02).

#### Psychological variables: Anger and externalising problems

Disruptive behaviour and anger were assessed by Hartwig [73] using the Beck Disruptive Behaviour Inventory (BDBI) and Beck Anger Inventory (BANI). There were 15 children in the HART+AAT and 14 children in the HART only group. Although all children showed significant reductions in disruptive behaviour, the difference between the two groups in rate of change was not significant. In terms of anger, no significant differences were seen over time and between groups.

Hanselman [76] measured anger on the State-Trait Anger Scale (STAS-TAS) and by a subjective mood thermometer. The author reported a significant decrease in the mean state anger (STAS) and trait anger (TAS) from pre to post intervention and an increase in the mean rating for subjective anger for beginning to end of sessions. Statistical analyses were not reported.

Stefanini, Martino [72] assessed externalising symptoms via the Youth Self-Report (YSR). There was no significant difference between the control group (TAU) and the intervention group (TAU + AAT) with regard to rate of change to externalising symptoms.

#### Psychological variables: Adaptive functioning, wellbeing, coping & self-concept

Subjective wellbeing and coping was assessed by Hamama, Hamama-Raz [74] by asking “All things considered, how satisfied are you with your life these days?” and by asking the participant to rate her perceived coping with stressful or extreme events. Responses were rated on a 4-point Likert scale. No significant change was reported pre- to post-intervention (*p* = .447, and *p* = .594 respectively). Relative to the comparison group (without trauma history), the intervention group had a lower level of subjective wellbeing pre-intervention (*p* = .041) that was not present post-intervention (*p* = .116). Hartwig [73] assessed self-concept with the Beck Self-Concept Inventory (BSCI). There were no significant differences pre to post-test for either the intervention group (HART + AAT) or the comparison group (HART).

Stefanini, Martino [71] explored the adaptive functioning of participants via a number of staff-reported (not facilitators) measures including; The Children’s Global Assessment Scale (C-GAS), format of hospital care (indicating the clinical severity of the patient on a 3-point scale), and ordinary school attendance (capacity for school attendance from 1, hospital school only, to 3, regular attendance at ordinary school). The intervention group (TAU + AAT) made significant improvements over and above the control group (TAU) in global functioning (*p* < .001), format of hospital care (*p* = .020) and ordinary school attendance (*p* < .030). Similar results were found by Stefanini, Martino [72]; there was a significant improvement to global functioning on the C-GAS from pre- to post-intervention (*p* < .001) and a significant improvement when compared to the control group (rate of change) (p < .001). The intervention group had significantly greater improvement on the total competence scale of the YSR (*p* = .001).

#### Psychosocial variables: Engagement, socialisation & connection

Thematic analysis conducted by Lubbe and Scholtz [56] of documents from the therapeutic case file (including a book made by the participant that contained letters, drawings, paintings and photos) and follow-up interview, identified five themes arising from the presence of the therapy canine: facilitating relationship building; enabling communication by working indirectly; experiencing physical affection through the therapy canine; socialisation skills; and enhanced self-esteem. Lange, Cox [75] similarly used qualitative methodology (semi-structured interview). There was too little data to conduct thematic analysis, but common topics identified were that canine presence was beneficial for: humour; calming; attendance; disclosure; self-soothing; and feeling attached.

Hanselman [76] reports informal, in-session observations made by the facilitators, indicating that positive behaviour increased when the canines were present. The author also reports an increase in the mean score on the Companion Animal Bonding Scale (CABS) pre-session to post-session, although statistical data is unavailable. Behavioural coding of videotaped sessions conducted by Stefanini, Martino [71] indicate significant increases in observed participation (*p* < .001), interaction (*p* < .001) and affection (*p* < .001) towards the canine, socialisation with adults (*p* < .001) and peers (*p* < .001), and reduced withdrawal (*p* < .04). Similar findings were reported by Stefanini, Martino [72]; significant increases in motivation (*p* < .001), relationships (*p* < .05), socialisation (*p* < .01) and affect (*p* < .001).

### Acceptability and dropout, tolerability, and feasibility

Limited information about acceptability, tolerability or feasibility is provided in the studies. With regard to acceptability (i.e., attendance and attrition), no drop-outs are evident or reported for four of the studies [56, 73, 74, 76]. Stefanini, Martino [71] and Stefanini, Martino [72] report 100% attendance rates to sessions for the intervention group, whereas Lange, Cox [75] reports that two of five participants were ‘unavailable’ for follow-up interview with no cause provided. Another potential measure of acceptability is enrolment rates. This can be estimated by calculating the number of eligible participants against the number of actual participants. The number of eligible participants is reported only by Hamama, Hamama-Raz [74], with nine girls consenting to participate in the intervention group out of a possible 20. It is not clear from the data if the majority of participants declined to participate due to concerns about acceptability, or other intervening factors, such as scheduling.

Tolerability (i.e., intended versus unexpected or unintended consequences) is not directly reported in four of the studies [71, 72, 74, 75]. An increase in depression was reported by Hanselman [76], attributed to increased emotional awareness and reduced substance use. Lubbe and Scholtz [56] imply that tolerability is high as the participant achieves positive outcomes in the AAT intervention where previous interventions were unsuccessful.

Lange, Cox [75], Lubbe and Scholtz [56] and Hartwig [73] reported on limitations to feasibility, stating that access to appropriately trained animals and handlers must be considered in addition to facilitator training and supervision. Assessment of the canine and liability issues must also be addressed [75]. Hartwig [73] further states that there were some difficulties adapting the HART curriculum to include the canines.

## Discussion

This review was the first attempt to synthesise the CAP literature, using clear definitions to delineate goal-focussed mental health treatments from informal activities that did not contain any psychotherapeutic theories or techniques. This review was also the first systematic review of AAT for participants in the distinct developmental period of adolescence. The characteristics of CAP interventions were explored, including the nature of interactions between participants, facilitators and canines in an attempt to determine the specific components of importance. The impact of CAP on primary mental health symptomatology was reviewed and distinguished from secondary factors associated with improved therapeutic processes and participant wellbeing. This resulted in a more comprehensive exploration than previous reviews on AAIs. Acceptability, tolerability and feasibility were also examined. These factors have not successfully been explored previously in the CAP literature.

### Intervention characteristics

Limited detail about interventions was provided in the studies reviewed. Based on the information presented, replicability of the reviewed studies is not possible. Although some authors included brief program outlines, only one intervention was reported to be standardised [73]. No authors discussed the temperament or behaviour of the canine or canines employed in the intervention. Given previous findings indicating that spontaneous engagement with a boisterous and puppy-like Collie was more effective at reducing depression than facilitator-led and structured interaction with a quiet Collie [40], further exploration of canine temperament and behaviour, is needed.

Hartwig [73] discussed the difficulties of adapting existing curriculum to include canines, reflecting that active and experiential activities may have been more therapeutic. Such experiential interactions are well documented in models of equine-assisted interventions [77–82], but are not evident in canine-assisted interventions. It will be important for future reviews to explore the differences between heavily structured, facilitator-led interventions and those that allow for spontaneous and participant-led interactions before conclusions can be drawn about the efficacy of various models of engagement.

Three studies indicated that the participant-canine interactions were the central tenet of the therapy, that is, all of the therapeutic content was centred around canine-related activities [71, 72, 74], whereas in others the canine was present only for portions of the therapy [76]. This is important to explore further, as meaningful integration of canines into the therapeutic process has been shown to increase efficacy [41].

Integration between theories of human-animal engagement and those for psychological treatment were poorly reported. Only two studies included information about existing psychological theories or techniques that informed the nature of the intervention, such as play therapy, attachment theory, solution-focused therapy and CBT [73, 76]. The remaining interventions were informed by research indicating that the presence of a friendly animal can facilitate engagement or rapport, reduce arousal or anger, and increase socialisation, all of which may be beneficial to the treatment process.

Various intervention formats were utilised, including individual counselling, group counselling, and combinations thereof. Dosages spanned 8.3 total hours [73] to 36 total hours of treatment [74]. A range of settings were reported, including inpatient and community, outdoor and indoor. Whilst previous reviewers have noted that format (group), dose, or location (indoors/outdoors) may impact outcomes [11, 14], this review is inconclusive. There is also evidence to suggest that simply being in nature and outdoors environments can improve mental health [83, 84]. To date the impact of nature (being outdoors) has not been separated from the impact of animal species [14, 85].

### Impact of CAP on primary diagnosis and clinical symptoms

There is preliminary evidence to suggest that the inclusion of canines in mental health treatments for adolescents may confer additional benefits to primary clinical symptomatology, that is, to the symptom or condition that is the primary target of the intervention. The impacts, however, were not universal. The two studies that used an RCT design provided promising results, indicating a reduction in clinical symptoms over and above treatment as usual for global functioning and internalising (emotional) problems [71, 72]. Externalising (behavioural) problems improved but not significantly more than the control group [72].

Studies conducted with lower levels of methodological rigour reported mixed findings, indicating that CAP may have been helpful in reducing some symptoms over and above treatment as usual, namely PTSD symptoms [74], but not others, including depression, anxiety and disruptive behaviour over the standard treatment without the canines [73, 74]. One further study reported on clinical symptoms [76], but the study was of such poor quality, that any conclusions must be treated conservatively and with caution. The intervention, an anger management group, was associated with reductions in emotional and behavioural anger. The author also reported an increase in depression that was attributed to greater emotional awareness and reduced substance use [76].

There is no evidence to suggest that the inclusion of canines in adolescent mental health treatments will increase the impact on clinical symptoms that are not the subject or focus of the therapy. For example, Hartwig [73] reports no improvements to anger; however, anger was not a treatment goal for any of the study participants. There is a general perception in the media and the community that visitation with a canine can improve the symptoms of mental illness without the inclusion of targeted therapy [6]. This perception is not supported by the current review.

### Impact of CAP on secondary factors, including therapeutic processes & participant wellbeing

Participant feedback, formal and informal observations indicate that CAP has positive impacts on engagement and socialisation behaviours, even when these have not been the primary focus of the intervention. Positive, pro-social engagements such as talking and showing affection were reported by a number of the authors [56, 71, 72, 75, 76] and are consistent with the existing literature [38, 39]. These are factors that may have an important impact on the process (and hence outcome) of therapy, such as developing collaborative relationships [52], or maintaining a strong therapeutic alliance [86]. Qualitative reports also indicate a reduction in negative and disruptive behaviours displayed during interventions [75, 76].

These findings may be a product of the canine’s ability to increase calm, self-soothing and feelings of attachment [38, 39, 56, 75, 76, 87], and provide further support for the oxytocin hypothesis, which proposes that in the presence of a friendly animal, humans produce the attachment promoting and anxiolytic hormone oxytocin, resulting in increased pro-social and decreased anti-social interactions [53, 88].

There is insufficient evidence from this review to conclude that CAP will improve factors associated with participant wellbeing; such as self-concept, self-esteem, coping or subjective wellbeing [73, 74], with only one case study finding self-reported improvements to self-esteem [56]. This finding is despite a general perception that working with canines can increase self-efficacy or mastery [56, 74]. There is, however, evidence to support the finding that CAP may improve global functioning over and above typical treatments [71, 72].

### Acceptability, tolerability and feasibility of CAP

Based on the current literature review, CAP has high attendance and retention rates for adolescents with mental health concerns, indicating high acceptability of CAP compared to standard psychological treatments. Stefanini, Martino [71] and Stefanini, Martino [72] both reported 100% attendance rates to each treatment session of their interventions. Although the data was poorly reported, no dropouts are evident or reported for the therapeutic component of the seven interventions reviewed. If accurate, this speaks to high acceptability of these interventions. This is important, as attrition is expected in mental health populations [89, 90] and is closely linked to engagement [91]. Findings were supported by qualitative feedback indicating that CAP improved attendance, disclosure, relationships and communication [56, 75]. This is in line with existing literature that suggests that the inclusion of a canine into psychotherapy improves therapeutic alliance, trust and willingness to disclose [37, 55]. It was not possible to establish from this review how likely adolescents would be to engage with CAP in comparison to other treatments. Given the importance of engagement in this population group, these are key areas for further study.

Tolerability of CAP, based on the current findings, appears satisfactory for this population. The majority of studies reported intended positive outcomes [56, 74, 75] including those that were statistically superior [71, 72] or equivalent to [73] existing psychological treatments. Only one study reported increased depression that was not attributed to the treatment [74].

CAP requires additional resources over and above traditional treatments, impacting feasibility in some settings. For example, access to large rooms and outdoor spaces, may be of benefit [71–74]. Facilitator training and/or supervision is required [73, 75], even when employing external canine and handler teams [71, 72]. Screening, training or assessment of the canines is also required [71–73, 75]. In this review, no authors reported on the welfare of the canines, despite repeated calls in the literature to do so [92]. This is an area which requires additional awareness and attention.

### Limitations

This review was limited by the small number of studies (seven), their heterogeneity, and resultant inability to perform meta-analysis. Only four of the studies achieved ‘fair’ or ‘good’ methodological quality [65, 66] plus a moderate to high level of evidence [1, 69]. The details of interventions were poorly reported, precluding replicability. There was significant variance in the nature of the interventions, meaning that this review was unable to report on the impact of elements within the interventions such as location, format, dose or role of the canine/facilitator/handler. Nevertheless, this review contributes preliminary evidence to support the inclusion of canines into mental health treatments for adolescents, to improve primary clinical symptoms, facilitate attendance, increase rapport and positive socialisation. Including a canine into therapy may also improve acceptability and tolerability for this difficult to engage population group. Importantly, all of the studies engaged voluntary participants who consented to be involved in interventions that included canines. This may have resulted in a self-selection bias where conclusions are only generalizable to those adolescents who like canines and would readily consent to engage with canines as a component of their mental health treatments.

### Recommendations and future directions

A number of questions have arisen from the current literature namely, what are the specific interactions or elements that create change and for which participants? Some excellent recommendations to improve the quality of the literature have been made in previous reviews including: clear and accurate use of terminology to describe interventions, such as those developed by the IAHAIO [5]; ensure therapeutic interventions are distinguished from brief unstructured activities [14, 26]; and calls for standardization (manualisation) of interventions to improve treatment fidelity and replicability [15, 26]. Whilst we support and reinforce these recommendations, there are a number of very promising lines of enquiry that should be adopted in future research.

#### An exploration of canine-facilitator-participant engagement

First, an exploration of the engagement between facilitators, participants and canines is crucial. Previous literature indicates that simple canine-presence is sufficient to impact treatment outcomes [34], whereas other authors have indicated that active engagement [73] or matching of canines to participants [71, 72, 74] may also be important. Crucially, relevant and meaningful canine-presence that is clearly related to the psychotherapeutic content has been found to have a superior effect to incidental presence [41] and warrants further research. The role of the temperament or behaviour of the canine should also be examined, to explore what, if any, impact this has. Previous literature has indicated that there may be a difference between canines who do and do not solicit affection and attention [40]. Obtaining information from participants about the nature of their relationship with the canines, and their perception of the canine’s role within the therapy is an important future step for authors; specifically, how engaging were the canines, how meaningful was their presence, and how relevant were the canines to the content of the therapy. To our knowledge, no standardised measures exist to explore these factors, and hence qualitative sampling should be used to elicit this information.

There have been many calls within the field for understanding of the characteristics of interactions and interventions that cause change. To date, there have been no clear methodologies by which to do this. There have been some attempts to describe the level of ‘structure’ that occurs in AAT upon a continuum, from non-directive play therapy through to greater structure that occurs when teaching a canine a trick [93]. This concept has not been reflected in any of the studies we reviewed. We therefore recommend development and application of a consistent nomenclature to describe the nature of the engagement with animals (of all species), and also the role of the facilitator and participant in that engagement.

**Spontaneous interactions** are those in which the animal and the participant engage naturally. In these interactions the animal is free to respond instinctively to the state or behaviour of the participant(s), such as those applied by White, Quinn [39], and can be referred to as animal-led. Spontaneous interactions provide opportunities for the development of insight, as the canine reflects to participants their level of arousal or emotional state, and these may be interpreted by the facilitator (handler) [94]. They also allow for the development of a meaningful relationship to form between the participant and the canine, outside of their relationship with the facilitator [8].

**Adjunctive interventions**, such as those employed by Hartwig [73], encompass structured and semi-structured interventions in which the facilitator guides the interactions between the animal and the participant, in order to achieve a pre-determined outcome. They are facilitator-led, and ‘in addition to’ the standard treatment activity. For example, teaching a canine to sit in order to develop communication or social skills, or engaging a canine in a mindfulness exercise. Having animals present during these interactions may improve motivation to participate [95], or salience of the learned materials [96–98].

**Experiential interventions** do not appear to have been used widely within the canine-assisted literature but are prevalent in the equine-assisted literature. It does not appear that experiential interventions were utilised in any of the studies included in this review; although, Hartwig [73] reflected that their use may have improved treatment outcomes. Experiential interventions include interactions where the environment is set up by the facilitator, who then takes a non-directive or supportive role during the interaction, allowing the participant to practice or experience change together with the animal, thereby learning by ‘doing’. These interventions are best described as participant-led. For example, the facilitator may ask a participant to design an obstacle course that reflects their current challenges, and then invite the canine to navigate the obstacles with them, without help from the facilitator or canine-handler. Participants may be invited to ‘walk with’ or ‘play with’ the canine in whatever way feels right for the situation. Upon reflection the participant is encouraged to develop their own meanings from the interactions.

Application of a language to describe the nature and types of interactions that are occurring during interventions, together with standardisation of the interventions will lead to a greater ability to evaluate the role of animals, facilitators and participants and assess their unique contributions.

#### Theoretical foundations

Second, we recommend that further attention is given to the links between the theories of human-animal interaction, for example the oxytocin hypothesis [99], and how this relates to the nature or type of interventions that are being conducted within therapy [53]. This review provides evidence to suggest, for example, that canine presence may be particularly beneficial to motivate attendance and improve retention [71, 72] possibly via the release of oxytocin in participants [53]. These benefits may be of particular importance in the early phases of therapy in order to assist with participant engagement. This information may be utilised to ensure engagement-related activities are made a priority at the beginning of treatment.

This review also supports the existing literature which suggests that animal presence may be soothing, calming or stress-relieving [34] possibly via reductions in stress physiology [53]. A focus on canine-related calming activities may therefore be particularly useful during the middle and later phases of therapy when participants may be addressing difficult or distressing content–such as trauma processing, and require assistance with grounding, mindfulness or distress tolerance. Similarly, researchers and facilitators have previously identified the ability of canines to give non-verbal feedback with regard to the state or behaviour of participants [7, 8, 94]. This may be particularly beneficial during the early and middle phases of therapy when there is a focus on the development of self-awareness, behavioural or emotional regulation. Working with canines may therefore provide real-life opportunities to practice cognitive and behavioural reframing. It is also important for participants to feel confident in their ability to maintain gains or changes once they leave therapy. The development of healthy relationships, and the internalising of new skills are therefore important elements in the final phases of therapy. Encouraging increasingly participant-led and experiential interactions during the final phases of therapy may allow the participants to develop increased confidence in their abilities, and the capacity to practice skills with someone other than the facilitator (i.e., with the therapy canine).

To date, we have been unable to find any peer-reviewed, published canine-assisted therapy protocols which draw on these theoretical foundations. Making explicit theoretical links between the content of therapy and the types of interventions and interactions chosen will give greater credibility to the field, distinguishing canine-assisted psychotherapy from other canine-related activities.

#### Assessment of standardised interventions

Third, it is important to assess the outcomes of any standardised CAP interventions and make changes accordingly. There have been numerous calls within the literature to increase the number of high quality randomised controlled trials (RCTs) of AAT [e.g. 10]. It is not clear, however, what type of AAT should be trialled, nor the important elements to include in such an intervention. There are currently no published, clearly defined or recognised CAP protocols which could form the basis for RCTs. We therefore recommend that following the development of a standardised protocol, preliminary trials are conducted to assess acceptability, tolerability, feasibility and qualitative outcomes, prior to moving forward into RCTs.

## Conclusions

There is evidence to suggest that CAP may improve the efficacy of mental health treatments in self-selected adolescent populations via reductions in primary symptomatology including PTSD and internalising symptoms, and the severity of serious psychiatric disorders. CAP may also confer additional benefits via secondary factors that improve therapeutic processes and quality, such as attendance and retention, positive socialisation, and feelings of connection.

Much work still needs to be done to establish the key components of CAP interventions that are most effective. The authors of this review have proposed a clear nomenclature to describe the interactions between canines, facilitators and participants, and provided recommendations for establishing theoretically grounded, standardised (manualised), CAP intervention protocols that may subsequently form the basis of efficacy and effectiveness testing via RCTs.

We received no funding for this review.

## Supporting information

S1 Fig PRISMA 2009 checklist. (DOC)

S1 Table Database search terms. (DOCX)
