# Supplementary material for: Incorporating animal-assisted therapy in mental health treatments for adolescents: A systematic review of canine assisted psychotherapy
Source: PLoS One. 2019 Jan 17;14(1):e0210761. doi: 10.1371/journal.pone.0210761 (PMC6336278; doi:10.1371/journal.pone.0210761)
Supplement: S1 Table — (DOCX) [file pone.0210761.s002.docx]

**S1 Table. Database Search Terms**

| Database | Search terms |
| --- | --- |
| PsycINFO | 1. ((animal or dog* or canine or non-human or pet or human-animal or animal-assisted) not rat* not rodent* not equine* not horse* not mice not mouse not ape* not monkey* not fish not bird not chimpanzee* not animal model* not animal stud* not chicken* not animal find*).mp. [mp=title, abstract, heading word, table of contents, key concepts, original title, tests & measures]  2. (counselling or counseling or intervention or learning or psychotherapy or therap* or mental* or emotion* or behaviour* or behavior* or affect* or empath* or trauma*).mp. [mp=title, abstract, heading word, table of contents, key concepts, original title, tests & measures]  3. (youth or young person or teen* or adolesc* or child*).mp. [mp=title, abstract, heading word, table of contents, key concepts, original title, tests & measures]  4. 1 and 2 and 3 |
| MEDLINE (Ovid) | 1. ((animal or dog* or canine or non-human or pet or human-animal or animal-assisted) not rat* not rodent* not equine* not horse* not mice not mouse not ape* not monkey* not fish not bird not chimpanzee* not animal model* not animal stud* not chicken* not animal find*).mp. [mp=title, abstract, heading word, table of contents, key concepts, original title, tests & measures]  2. (counselling or counseling or intervention or learning or psychotherapy or therap* or mental* or emotion* or behaviour* or behavior* or affect* or empath* or trauma*).mp. [mp=title, abstract, heading word, table of contents, key concepts, original title, tests & measures]  3. (youth or young person or teen* or adolesc* or child*).mp. [mp=title, abstract, heading word, table of contents, key concepts, original title, tests & measures]  4. 1 and 2 and 3 |
| Scopus | TITLE-ABS-KEY ("animal" OR "dog*" OR "canine" OR "non-human" OR "pet" W/3 "therapy" OR "psychotherapy" OR "learning" OR "intervention" OR "counselling" OR "counseling" OR "mental*" OR "therap*") AND ("youth" OR "young person" OR "adoles*" OR "teen" OR "child") SCOPUS SEARCH = 902; LIMIT TO ENGLISH; LIMIT TO ARTICLE (DOCUMENT TYPE); LIMIT TO JOURNALS (SOURCE TYPE). |
| Web of Science | TS = (animal or dog* or canine or non-human or pet or human-animal or animal-assisted) not rat* not rodent* not equine* not horse* not mice not mouse not ape* not monkey* not fish not bird not chimpanzee* not animal model* not animal stud* not chicken* not animal find*).  TS = (counsel* or intervention or learning or psychotherapy or therap* or mental* or emotion* or behaviour* or behavior* or affect* or empath* or trauma  TS = (youth or young person or teen* or adolesc* or child*).  4. 1 and 2 and 3 |
